# An interdisciplinary approach to improving conservation outcomes for parasites

**DOI:** 10.1111/cobi.14431

**Published:** 2025-01-19

**Authors:** Timothy M. Brown, Alison M. Dunn, Rupert J. Quinnell, Ellen Clarke, Andrew A. Cunningham, Simon J. Goodman

**Affiliations:** ^1^ School of Biology, Faculty of Biological Sciences University of Leeds Leeds UK; ^2^ School of Philosophy, Religion and History of Science, Faculty of Arts, Humanities and Cultures University of Leeds Leeds UK; ^3^ Institute of Zoology Zoological Society of London London UK

**Keywords:** coextinction, conservation philosophy, conservation prioritization, conservation social science, ex situ conservation, holobiont conservation, parasitology, underappreciated biodiversity, biodiversidad infravalorada, ciencias sociales de la conservación, coextinción, conservación ex situ, conservación holobionte, filosofía de la conservación, parasitología, priorización de la conservación

## Abstract

Parasites represent a significant proportion of Earth's biodiversity and play important roles in the ecology and biology of ecosystems and hosts, making them an important target for conservation. Despite increasing calls to prioritize protection for parasites in the academic literature, they remain undervalued and underrepresented in global biodiversity conservation efforts, not least due to the perception that the interests of parasite and host conservation are opposing and the common misconception that parasites are a threat, rather than a benefit, to conservation. We considered whether taking an interdisciplinary approach to parasite conservation research will generate novel insights and solutions concerning why and how parasite conservation should be practiced for the benefit of parasites, their hosts, ecosystems, and people. We argue that 2 of the main barriers to more widespread parasite conservation are the knowledge gap concerning the role of sociocultural factors affecting the willingness to enact parasite conservation and the lack of a consistent and cohesive philosophical basis for parasite conservation. Possible sociocultural barriers to parasite conservation include misconceptions of the risks posed by parasites, taxonomic bias, differences in conservation values, economic constraints, and technical challenges. The use of social science can generate insights into levels of awareness and support for parasite conservation and improve understanding of how human values and attitudes mediate conservation practices concerning parasites. Such knowledge will have a critical role in addressing sociocultural barriers and improving support for parasite conservation. Issues with the current philosophical basis for parasite conservation include contradictory accounts of which parasites merit conservation, insufficient explanation of how different conservation values apply to parasite biodiversity, and the existence of a false antagonism between host and parasite conservation. Greater engagement with philosophical work on environmental ethics and biological unitization will strengthen existing arguments for parasite conservation and will support conservation decision‐making processes.

## Abstract (es)

Un enfoque interdisciplinario para aumentar los resultados de conservación para parásitos

**Resumen:**

Los parásitos representan una parte importante de la biodiversidad de la Tierra. También tienen un papel importante en la biología y ecología de los ecosistemas y sus hospederos, por lo que son un objetivo importante para la conservación. A pesar de que en la bibliografía científica hay cada vez más peticiones para priorizar la protección de los parásitos, todavía están infravalorados e infrarrepresentados en los esfuerzos mundiales de conservación de la biodiversidad. Esto se debe sobre todo a la percepción de que los intereses de la conservación de parásitos y hospederos son contrarios y la creencia común de que los parásitos son una amenaza y no un beneficio para la conservación. Contemplamos si usar un enfoque interdisciplinario en la investigación sobre la conservación de parásitos generará información y soluciones con respecto a cómo y por qué se debería realizar la conservación de parásitos en beneficio de los mismos, sus hospederos, ecosistemas y las personas. Sostenemos que dos de las principales barreras para una conservación más amplia de los parásitos son las lagunas de conocimiento con respecto a los factores socioculturales que afectan la voluntad de llevar a cabo esta conservación y la falta de una base filosófica uniforme y coherente para la conservación de parásitos. Las posibles barreras socioculturales para la conservación de parásitos incluyen la creencia errónea del riesgo que representan los parásitos, el sesgo taxonómico, las diferencias en los valores de conservación, las restricciones económicas y los obstáculos técnicos. Las ciencias sociales pueden brindar información sobre los niveles de sensibilización y respaldo para la conservación de los parásitos y también mejorar el entendimiento de cómo los valores y las actitudes humanas interceden en las prácticas de conservación correspondientes a los parásitos. Dicho conocimiento tendrá un papel importante en cómo se abordan las barreras socioculturales y en el incremento del respaldo para la conservación de parásitos. Los temas con la base filosófica actual de la conservación de parásitos incluyen los testimonios contradictorios sobre cuáles parásitos merecen ser conservados, la falta de explicaciones sobre cómo los valores diferentes de conservación aplican para la diversidad de parásitos y el antagonismo falso entre la conservación de hospederos y la de parásitos. Una mayor intervención de trabajos filosóficos sobre la ética ambiental y la utilización biológica fortalecerá los argumentos existentes a favor de la conservación de parásitos y respaldará los procesos de toma de decisiones en la conservación.

## INTRODUCTION

The global extinction crisis is widely accepted as one of the greatest challenges facing humanity (Cowie et al., 2022; WWF, 2022). In response, significant amounts of economic resources and human effort have been mobilized in attempts to prevent further biodiversity loss and to mitigate a sixth mass extinction event (Waldron et al., 2017). However, little attention has been given to the extinction of dependent species, including parasites, which are likely to constitute a majority of species extinctions over the next hundred years (Dunn et al., 2009). To address this gap, we propose an interdisciplinary approach to parasite conservation research to improve outcomes for the conservation of parasites, their hosts, and ecosystems.

The roles that parasites play in ecosystems are highly underappreciated. Without parasites, ecosystems become less diverse, less productive, and less resilient (Colwell et al., 2009; Wood & Johnson, 2015). Parasites are also overlooked for their roles in host evolution and immune system functioning, both of which support increased chances of survival at a population and individual level (Coltman et al., 1999; Van Oers et al., 2002). In many cases, parasites are simply disregarded by biodiversity conservation efforts, and there has been speculation, but little empirical investigation, about the rates of parasite species extinctions and coextinctions resulting from declines in host abundance and from host species extinctions, respectively (Colwell et al., 2012). Coendangered parasite species may also be more directly driven toward extinction by conservation activities that focus solely on the host species. This contradiction is demonstrated by the case of the black‐footed ferret louse (*Neotrichodectes minutus*), which was eradicated from its host by insecticide treatments applied to prevent the transmission of sylvatic plague by fleas during the host captive breeding program in the 1980s (Gompper & Williams, 1998). *Neotrichodectes minutus* has been presumed extinct ever since (Harris et al., 2014). Whether the extinction of a parasite species is viewed as a conservation failure or a successful eradication effort depends on the point of view of the people involved.

In response to the loss of parasite biodiversity, a growing number of conservationists have called for the consideration of the conservation of parasite species alongside that of their host taxa. The concept of parasite conservation was introduced by Windsor (1990). At first, parasitologists drew attention to the heightened threat level of many parasite species (Durden & Keirans, 1996; Stork & Lyal, 1993) and suggested that parasites should be recognized as equally valid targets of conservation as their host organisms (Gompper & Williams, 1998; Windsor, 1995). Since then, parasite conservation has developed into a broad research area, spanning parasitology, ecology, and conservation biology, and centered around the need for a paradigm shift in how conservation is conceptualized and practiced with regard to parasites (Dougherty et al., 2016). Parasite conservation research encompasses studying the reasons parasites need to be conserved (grounded in macroecology and evolutionary processes), factors influencing parasite extinction risk and its distribution among different parasite taxa, and practical approaches to parasite conservation (Carlson et al., 2020).

Despite the increasing academic interest in parasite conservation and proposals for parasite conservation frameworks (Carlson et al., 2020; Dougherty et al., 2016), successful interventions remain limited in practice. Few parasites have systematic conservation plans, and where these do exist, they are rarely implemented and often limited in taxonomic and geographic scope. Among the positive examples are conservation plans for 2 host‐specific ectoparasite species of Iberian lynx (*Lynx pardinus*) and Manx shearwater (*Puffinus puffinus*) (Kwak et al., 2019; Perez et al., 2013) and provision for the coconservation of parasites in the Tasmanian devil recovery plan (Wait et al., 2017). In general, however, parasites are typically either ignored by conservationists or, where they are considered, assumed to be a threat to host species rather than a target for conservation in their own right (Gómez & Nichols, 2013). As a result, there exists a critical gap between arguments outlining the ecological importance of parasite conservation and the uptake of parasite conservation approaches by practitioners. The reasons behind this theory–practice disconnect are poorly explored; there is a particular lack of knowledge on how sociocultural factors influence conservation action. For example, we are aware of no in‐depth studies of cultural values and attitudes around parasite conservation. It is unclear how sociocultural factors influence practitioner decisions in relation to parasite conservation, including such factors as the level of awareness of the ecological importance of parasites or of parasite extinction risk, knowledge of parasite conservation interventions, taxonomic bias, misconceptions of the risks posed by parasites, and concerns about cost and technical challenges. Another potentially important factor is how conservationists attribute value to different elements of biodiversity and whether these values are consistently applied to both free‐living and parasite biodiversity. To close the gap between conservation theory and practice, it is important to understand whether the current rationale for parasite conservation is not widely known to conservationists or whether it is known but contested.

We argue that research on parasite conservation needs to embrace a broader range of disciplines to develop understanding of the social, cultural, and ethical factors influencing parasite conservation outcomes. Interdisciplinary research will help disentangle the factors influencing conservationists’ involvement with parasite conservation and improve understanding of and help in the resolution of conflicts over whether parasite biodiversity should be conserved. We reviewed current perspectives on parasite conservation, covering the evolutionary and ecological importance of parasites and different approaches to parasite conservation. We then evaluated how a social science approach could address the knowledge gap concerning how sociocultural factors mediate practices around parasite conservation. We also explored sociocultural factors that may account for the apparent lack of practice. We then critically examined several of the arguments currently given in favor of parasite conservation. We considered whether parasite conservation can benefit from incorporating philosophical approaches that can be used to evaluate different justifications for and against parasite conservation, including in cases where there may be opposing views. Overall, we considered a more interdisciplinary approach to parasite conservation research that would support the development of interventions that meet the diverse needs of humans, parasites, and free‐living biodiversity and thus improve conservation in the whole.

## HISTORY AND CONCEPTS AND APPROACHES TO PARASITE CONSERVATION

We use the term *parasite* to mean any organism that is dependent on living on or in a host organism for all or part of their life cycle and that derives resources at the expense of the host, with the potential to decrease host fitness (Hatcher & Dunn, 2011; Goater et al., 2014). This includes helminths, arthropods, protozoa, viruses, bacteria, and fungi, although the majority of parasite conservation research has focused on macroparasites (helminths and arthropods), rather than microparasites that might present a greater threat to human health and livelihoods or to host survival (Carlson et al., 2020). Parasite taxa represent a large proportion of all species on Earth (Dobson et al., 2008; Strona & Fattorini, 2014; Windsor, 1998) and act as a dominant force in food webs, where they are involved in a majority of species linkages (Lafferty et al., 2006) and are an important source of selective pressure on host species (Coltman et al., 1999; Dobson & Hudson, 1986). In its original conception, parasite conservation was proposed as a necessary response to taxonomic bias in biodiversity conservation efforts based on the argument that if conservation is interested in preserving biodiversity, this should also apply to parasite species, many of which are faced with a similar or even greater threat of extinction as their hosts (Windsor, 1990). This argument has been developed to reflect a wide spectrum of conservation values, ranging from the idea that parasites are instrumentally valuable as integral parts of ecosystems, to the notion that parasite species are intrinsically valuable and should be conserved for their own sake (Lymbery & Smit, 2023).

There are 2 different ways that parasites may be understood as being intrinsically valuable. The first is the view of subjective intrinsic value, in which value could be conferred to parasites by humans on the basis of what they are, rather than their use as a means to achieve a desired end. The second is the perspective of objective intrinsic value, where species are understood as valuable independent of their relationship to humans (Sandler, 2012). There are numerous reasons why one might subjectively value parasite species for themselves, including their evolutionary distinctiveness (Cardillo, 2023), their uniqueness and irreplaceability (Miller et al., 2007), or appreciation for their diversity of form and function (Dougherty et al., 2016). Although the subjective view of intrinsic value is generally more intuitive than the objective view, it may be hard to motivate a widely held sense of this value for parasites due to a lack of knowledge of their biology (Nichols & Gómez, 2011) and taxonomic bias (Pizzi, 2009). Objective intrinsic value is comparatively much harder to explicate than subjective intrinsic value, particularly for transorganismic entities, such as species and ecosystems. Although different accounts have been put forward attempting to identify a basis on which such entities might be considered objectively intrinsically valuable, independent of human valuation and beyond the value of their constituent parts, there remains no commonly accepted base property on which such value might exist (Sandler, 2012). Despite this, objective intrinsic value remains an attractive proposition because it avoids anthropocentrism and provides space for nonsentient objects to have a value of their own. Given the obvious commitment of conservation ecology to valuing and protecting various objects that are not sentient, including species, it is likely rash to give up on objective intrinsic value altogether. This may be particularly true in the case of parasite species given that objective intrinsic value, if substantiated, may provide a more stable basis for parasite conservation than subjective intrinsic value alone.

Parasites are under threat from many of the same extinction drivers that affect free‐living taxa, including climate change, pollution, and habitat loss (Carlson et al., 2017; Sures et al., 2023; Wood et al., 2023). However, due to their status as dependent organisms, parasites are also uniquely endangered by coextinction, the extinction of a symbiotic species following the extinction of its host species (Koh et al., 2004; Stork & Lyal, 1993). Many parasites rely on density‐dependent transmission, meaning that they are likely to go extinct before their hosts because they need access to larger host population sizes than their host populations require for persistence (De Castro & Bolker, 2005). Additionally, projections suggest that the coextinction of parasitic organisms will constitute the majority of species extinctions in the current extinction crisis (Dunn et al., 2009). The extinction risk of many parasites is further exacerbated by human actions involving the conservation of their hosts. Parasites are lost during ex situ host conservation either as the direct result of antiparasitic treatments applied to hosts or as an indirect consequence of removal from their environment and disruption to their life cycles, which may involve multiple host taxa or free‐living stages (Milotic et al., 2020).

In recent years, increasing importance has been placed on instrumental value in biodiversity conservation, as shown by the centrality of the ecosystem services concept in contemporary conservation debates, where nature is valued according to its utility for humans (Cornell, 2011). In parasite conservation research, there has been a renewed focus on the role that parasites play in ecosystem, evolutionary, and immune processes. At the ecosystem level, parasites alter food web dynamics, contribute to the flow of energy and nutrients throughout trophic networks, determine community and ecosystem structure by regulating host population abundance, and possibly increase ecosystem biodiversity (Hatcher et al., 2012; Hudson et al., 2006; Johnson et al., 2010; Kuris et al., 2008; Lafferty et al., 2006; Raffel et al., 2008; Sato et al., 2011). Parasites also play an important role in host evolution. Host–parasite coevolutionary dynamics can act to increase the genetic diversity of host populations (Coltman et al., 1999) and drive the evolution of host resistance to parasites (Alves et al., 2019; Harding et al., 2005; Schulte‐Hostedde & Mastromonaco, 2015). For host individuals, parasites are important for stimulating immune system development during early life stages (Spencer & Zuk, 2016). Moreover, host‐specific parasites may benefit host health by limiting the abundance of other, potentially more virulent, parasite species, although infection with one parasite can also make hosts more vulnerable to infection from other parasite species (Northover et al., 2018).

The loss of parasite biodiversity, and its associated roles in ecosystem function, individual host health, and host population dynamics, will result in ecosystems that are less complex, less productive, and less resilient (Colwell et al., 2009; Wood & Johnson, 2015). Thus, in terms of ecosystem services, a large proportion of the value that humans derive from ecosystems is indirectly derived from the activities of parasites. Humans can also derive direct benefits from parasites, as sources of biomedical compounds or nutrition (Overstreet, 2003; Wu et al., 2017). There is also scope for parasites to become important tools for host conservation because they can be used as bioindicators for ecosystem degradation and as bioinformation sources on host population and demographic history (Gagne et al., 2022; Gómez & Nichols, 2013).

With the value of parasite diversity becoming more widely appreciated, an important step in the development of parasite conservation is the identification of at‐risk species. Extinction risk assessments, such as those conducted by the International Union for Conservation of Nature (IUCN), guide effective conservation efforts (Rodrigues et al., 2006) and raise the public profile of endangered species (Cardoso et al., 2012). However, parasites seldom feature in threatened species lists. Only one animal parasite, the pygmy hog‐sucking louse (*Haematopinus oliveri*) (critically endangered), has been evaluated and listed on the IUCN Red List (Lymbery & Smit, 2023). Several different approaches have been proposed for addressing the lack of parasite extinction risk assessments. In 2023, the IUCN Species Survival Commission established a Parasite Specialist Group with the specific aim of increasing the number of parasites assessed by the IUCN (Hopkins & Kwak, 2023).

Alongside this, Kwak et al. (2020) proposed an alternative framework, the conservation assessment methodology for animal parasites (CAMAP), with the aim of improving the rate and accuracy of extinction risk assessments for parasites by addressing some of the limitations of the current IUCN Red List criteria when applied to parasites. For example, the abundance thresholds used by the IUCN are too low for parasitic invertebrates, which the CAMAP addresses by using the abundance of infected hosts as a surrogate measure for parasite abundance. The CAMAP has so far been used to evaluate the conservation status of the Manx shearwater flea (*Ceratophyllus fionnus*) (Kwak et al., 2019) and 3 species of host‐specific chewing louse found on crested ibis (*Nipponia nippon*) (Gustafsson et al., 2021). Although the CAMAP addresses several scientific barriers to parasite conservation assessments, the main challenge for increasing assessments is that they require research funding and taxonomic expertise, which are scarce for parasite diversity (Poulin, 2014).

After at‐risk parasites have been identified, conservation interventions can be carried out to mitigate further population declines (Table 1). The main goals for practical parasite conservation are the maintenance of parasite and host populations and the preservation of the environmental conditions required for parasite transmission from host to host. As such, conservation of threatened hosts will generally lead to the conservation of cothreatened parasites (Windsor, 2021). There are a limited number of studies assessing the benefits of in situ host conservation for parasite biodiversity. A meta‐analysis of the effects of fishing on parasite communities showed declines in parasite abundance and diversity as a result of declines in host species abundance (Wood & Lafferty, 2015), suggesting that marine protected areas are an effective strategy for conserving parasite biodiversity (Lafferty et al., 2008; Wood et al., 2013). Similar studies need to be repeated for terrestrial and freshwater realms and for a larger taxonomic range of hosts and parasites to ensure that in situ approaches are effective at conserving parasite biodiversity. However, host conservation will not always guarantee parasite conservation. It is possible for parasite species to go coextinct while their host species persist because parasites often require a higher host population density to ensure their viability than that required by hosts themselves. Dougherty et al. (2016) propose that parasites can be incorporated into host conservation by defining the minimum viable host population as that needed for associated parasites to persist. Parasites with complex life cycles also have more complicated conservation needs, requiring conservation of, and connectivity between, intermediate hosts and definitive hosts to ensure persistence.

**TABLE 1:** Selected examples of coendangered parasites for which conservation actions have been suggested.

Parasite	Host	Suggested conservation measure	Source
Arthropods			
Pygmy hog sucking louse, *Haematopinus oliveri*	Pygmy hog, *Porcula salvania*	Restricted use of antiparasitic treatments	Gerlach, 2014
Iberian lynx chewing louse, *Felicola isidoroi*	Iberian lynx, *Lynx pardinus*	Restricted use of antiparasitic treatments; sampling to develop knowledge of parasite biology and ecology; manual transfer of lice onto captive bred individuals; in vitro rearing of lice; International Union for Conservation of Nature assessment	Perez & Palma, 2001; Pérez et al., 2013
Black‐footed ferret louse, *Neotrichodectes minutus*	Black‐footed ferret, *Mustela nigripes*	Sampling to determine presence of parasite	Harris et al., 2014
Crested ibis chewing lice, *Ardeicola nippon*, *Colpocephalum nipponi*, *Ibidoecus meinertzhageni*	Crested ibis, *Nipponia nippon*	Sampling to improve knowledge of parasite biology and ecology; restricted use of antiparasitic treatments	Gustafsson et al., 2021
Wiradjuri flea, *Wurunjerria warnekei*	Leadbeater's possum, *Gymnobelideus leadbeateri*	Captive breeding of fleas on hosts or surrogate hosts; restricted use of antiparasitic treatments in situ	Kwak, 2018
Goblin flea, *Stephanocircus domrowi*	Leadbeater's possum, *Gymnobelideus leadbeateri*	Captive breeding of fleas on hosts or surrogate hosts; restricted use of antiparasitic treatments in situ	Kwak 2018
Plain thorny‐headed flea, *Acanthopsylla saphes*	Eastern quoll, *Dasyurus viverrinus*	Captive breeding of fleas on hosts or surrogate hosts; restricted use of antiparasitic treatments in situ	Kwak 2018
Hill's flea, *Pygiopsylla hilli*	Woylie, *Bettongia penicillata*; western ringtail possum, *Pseudocheirus occidentalis*	Captive breeding of fleas on hosts or surrogate hosts; restricted use of antiparasitic treatments in situ	Kwak 2018
Woodward's thorny‐headed flea, *Acanthopsylla woodwardi*	Western quoll, *Dasyurus geoffroii*	Captive breeding of fleas on hosts or surrogate hosts; restricted use of antiparasitic treatments in situ	Kwak 2018
New Holland flea, *Macropsylla novaehollandiae*	New Holland mouse, *Pseudomys novaehollandiae*	Captive breeding of fleas on hosts or surrogate hosts; restricted use of antiparasitic treatments in situ	Kwak 2018
Franklin Islands flea, *Acanthopsylla franklinensis*	Greater stick‐nest rat, *Leporillus conditor*	Captive breeding of fleas on hosts or surrogate hosts; restricted use of antiparasitic treatments in situ; cotranslocation	Kwak 2018
Manx shearwater flea, *Ceratophyllus* (*Emmareus*) *fionnus*	Manx shearwater, *Puffinus puffinus*	Sampling to improve knowledge of parasite biology and ecology; in situ protection including eradication of invasive species; translocation of parasite to establish insurance populations; public awareness campaigns	Kwak et al., 2019
*Satanicoptes armatus*	Tasmanian devil, *Sarcophilus harrisii*	Restricted use of antiparasitic treatments; sampling to establish parasite presence	Wait et al., 2017
*Diabolicoptes sarcophilus*	Tasmanian devil, *Sarcophilus harrisii*	Restricted use of antiparasitic treatments; sampling to establish parasite presence	Wait et al., 2017
Tuatara tick, *Amblyomma sphenodonti*	Tuatara, *Sphenodon punctatus*	Coreintroduction	Miller et al., 2007; Moir et al., 2012

Helminths			
*Neodiplostomum sarcophili*	Tasmanian devil, *Sarcophilus harrisii*	Restricted use of antiparasitic treatments; sampling to establish parasite presence	Wait et al., 2017
*Dasyurotaenia robusta*	Tasmanian devil, *Sarcophilus harrisii*	Restricted use of antiparasitic treatments; sampling to establish parasite presence	Wait et al., 2017
*Woolleya sarcophili*	Tasmanian devil, *Sarcophilus harrisii*	Restricted use of antiparasitic treatments; sampling to establish parasite presence	Wait et al., 2017

Various			
Various	Darwin's finches, *Thraupidae*	Sampling to establish parasite presence prior to use of antiparasitic treatments	Bulgarella & Palma, 2017

* All are threatened due to the endangerment of their hosts or conservation measures applied to their hosts.

A key consideration for parasite conservation is the threat that host conservation actions can pose to parasite extinction risk. Although there is a lack of systematic information on biosecurity and parasite treatment in ex situ conservation programs, anecdotal evidence suggests that parasites are commonly removed with little consideration of their conservation interest (Milotic et al., 2020). In response to this, parasite conservationists have argued for more judicial use of antiparasitic treatments during ex situ conservation (Kwak, 2018; Windsor, 2021).

To help address potential conflicts between host and parasite interests, Stringer and Linklater (2014) outline a set of principles for parasite control during host conservation, proposing that as the risk of host extinction increases, control methods with greater impacts on parasites are justified so long as parasites are provided with refugia if they are at risk of extinction. Parasite refugia can take a number of forms including transferring and maintaining parasites on alternative hosts until their main hosts have recovered (Gómez & Nichols, 2013) or rearing parasites in vitro (Gustafsson et al., 2021). Another approach is to maintain selected parasites of conservation interest on hosts, through manual transfer or via the use of narrow spectrum parasiticides, while still removing more harmful parasites (Pérez et al., 2013). Regardless of which approach to parasite conservation is used, it is critical that the interests of parasites are considered at the planning phase of host conservation.

Kwak (2018) coined the term *holistic conservation* to refer to conservation that recognizes the importance of species interactions and ecosystems for threatened species and aims to conserve these alongside host species. One way to do this is to shift the focus of conservation actions from individual species to species assemblages (Wait et al., 2017), for example, moving from reintroductions to coreintroductions to ensure that symbionts are relocated with their hosts and have their particular needs accounted for (Miller et al., 2007). An additional benefit of the species assemblage approach is that it prevents the need for multiple campaigns for each individual parasite species, reducing the likelihood of a kind of conservation fatigue setting in due to the sheer number of imperiled parasite species (Moir & Brennan, 2020). Notwithstanding the benefits of a species assemblage level approach, special care must be taken to ensure that the conservation needs of all codependents in the assemblage are met, including parasites with complex life cycles, which could create significant technical and economic challenges. Conservation programs managing multiple host–parasite systems should also adopt biosecurity practices to minimize risks of parasite contamination and maintain coevolved host–parasite communities (Fournié et al., 2015). Explicitly considering the needs of parasites during the planning stage of ex situ conservation is key to improving parasite conservation outcomes, alongside which monitoring of parasites after action will identify whether additional conservation actions are needed to support parasite persistence (Moir et al., 2012).

## SOCIOCULTURAL FACTORS AROUND PARASITE CONSERVATION AND THE NEED FOR A SOCIAL SCIENCE APPROACH

Despite academic interest in parasite conservation, practical interventions have been extremely limited, indicating a significant gap between parasite conservation in theory and practice. Even in the few cases where parasite conservation plans have been identified, they either have not been implemented (Kwak et al., 2019) or were identified too late, after it was no longer possible to implement them. For example, the black‐footed ferret louse (*N. minutus*) has not been observed since it was put forward as a potential flagship species for parasite conservation (Gompper & Williams, 1998; Harris et al., 2014). Accordingly, the best‐known successes of parasite conservation, whereby parasites have survived small population bottlenecks, have come from cases, such as the beaver beetle (*Platypsyllus castoris*) and 3 species of host‐specific lice of the crested ibis (*Nipponia nippon*), in which conservation was an unintentional side effect of host conservation (Gustafsson et al., 2021; Jørgensen, 2015).

A key barrier to current understanding of the apparent lack of parasite conservation is that there has been little systematic evaluation of how often conservation practitioners consider parasite conservation and how their decisions around it are influenced by sociocultural factors, including values, attitudes, and knowledge. With such a great knowledge gap, key challenges are to identify particular barriers that might be preventing concepts from being put into practice and to determine whether such barriers could be navigated to improve parasite conservation. This discussion sits within a wider context of the growing recognition of the importance of conservation social science, which understands conservation as a nexus of social and biological phenomena, directed by human subjectivities, that need to be critically examined and accounted for (Bennett et al., 2017).

**FIGURE 1:**
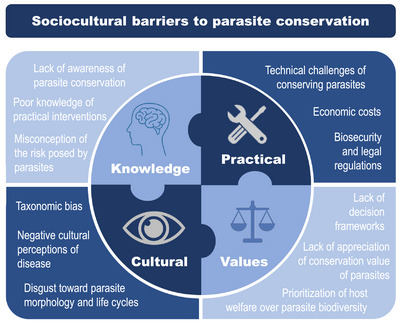
Potential sociocultural barriers to parasite conservation spanning categories centered on practitioner knowledge of parasite conservation, practical challenges, cultural influences, and value judgements.

To the best of our knowledge, there have been no systematic studies of the sociocultural determinants of the willingness of conservationists to engage with parasite conservation. We suggest that a number of factors could be important, including misconceptions of the risks that parasites pose; cultural factors, such as taxonomic bias; conservation value judgments, such as the prioritization of host welfare; and economic and technical challenges (Figure 1). At the most basic level, conservationists may simply be unaware of parasite conservation issues, including parasite extinction risk and conservation strategies. This is reflected in the almost total absence of parasites in academic conservation education, except when they are mentioned as a threat to hosts, meaning that conservationists are unlikely to perceive parasites as conservation targets (Nichols & Gómez, 2011). Similarly, conservationists may be overestimating the immediate risks parasites pose to their hosts and underestimating the risks of parasite eradication for hosts and ecosystems. Dougherty et al. (2016) draw a parallel between parasite conservation and predator conservation in the United States in the 20th century, suggesting that a paradigm shift is needed in which parasites go from being viewed purely negatively, as threats to health and the economy, to also being celebrated for their ecological importance.

**FIGURE 2:**
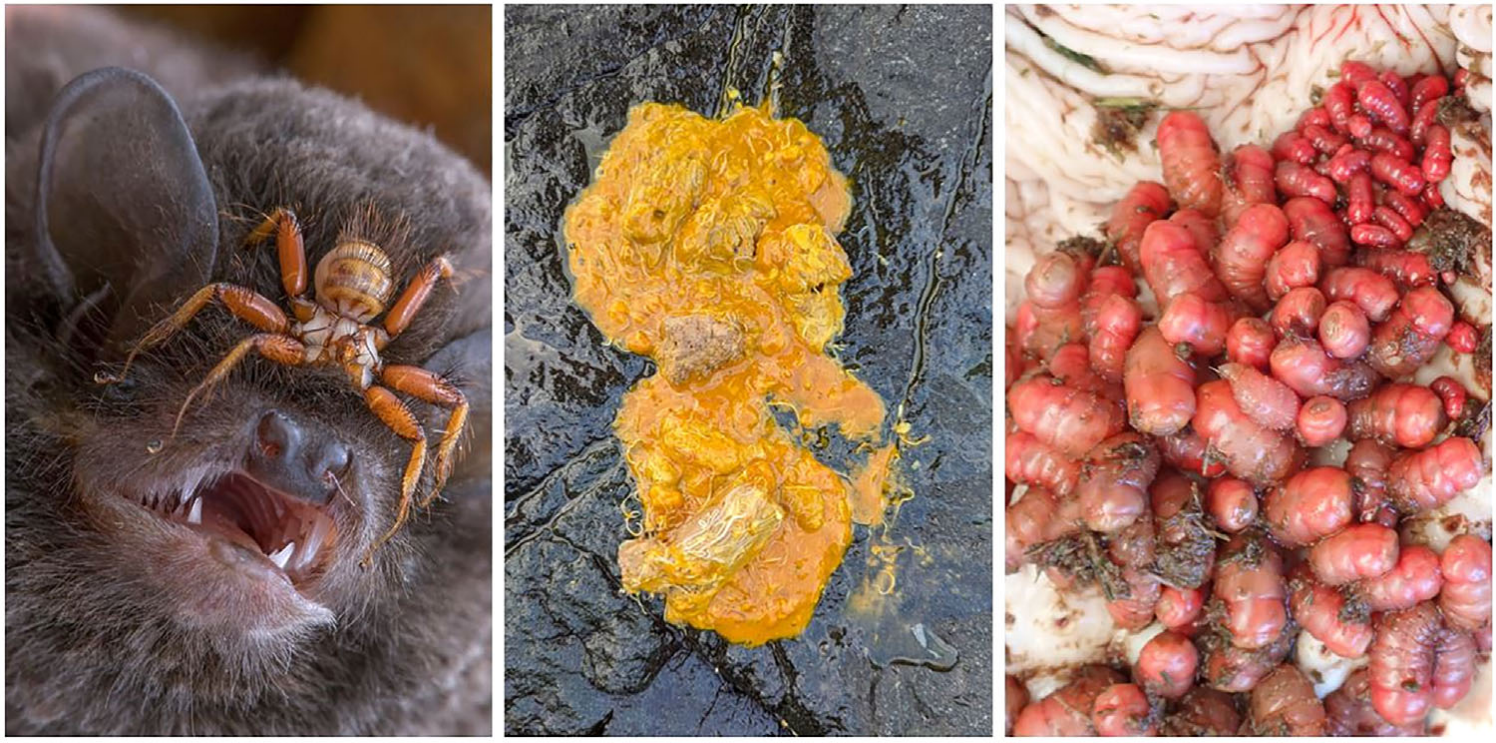
Overcoming human aversion to parasites and their life cycles will be a key part of gaining support for parasite conservation: (left) bat fly (Nycteribiidae) on a Mozambique long‐fingered bat (*Miniopterus mossambicus*) (photo by Piotr Naskrecki © [used with permission]), (centre) helminths in the feces of a gray seal (*Halichoerus grypus*) (photo by Simon Goodman ©), and (right) botfly larvae (*Gasterophilus* sp.) from the stomach of a mountain zebra (*Equus zebra*) (photo by Rupert Quinnell ©).

Culturally, it has been argued that parasites may be too taxonomically and morphologically different to humans for us to view them as valid targets for conservation (Simpson et al., 2024). Humans often exhibit taxonomic chauvinism, a bias against species at increasing taxonomic distance from us (Pizzi, 2009), and zoomorphic bigotry, an aversion to organisms with bodies unlike our own (Hatley, 2011), both of which could present significant barriers to parasite conservation (Figure 2). These biases are likely to be exacerbated by the innate feelings of disgust that humans display toward parasites and signs of parasite presence, which are a key part of an animal's ability to detect and alter behavior to reduce the risk of parasite infection (Weinstein et al., 2018). Similarly, cultural differences in hygiene practices could have a strong impact on the willingness of conservationists to take part in parasite conservation. Human biases could also be relevant if parasites eventually get conservation support, directing focus toward more charismatic species, or those that are technically easier or less costly to conserve, at the expense of more ecologically or evolutionarily important taxonomic groups (Isaac et al., 2007).

Conservation goals are also determined by how conservationists value different biological units, such as individuals, species, and ecosystems (Sandbrook et al., 2011). Conservation values could lead to low levels of engagement with parasite conservation on 2 counts. First, conservationists may be unaware that parasites possess many of the values according to which they conserve more charismatic species. Second, prioritizing individual host welfare at the expense of parasite conservation may represent a calculated value judgment that hosts are more valuable than parasites. This value judgment could be made across a number of different levels, from the decisions of individual practitioners to institutional, regulatory, and legislative requirements and guidelines. A key question is to understand the degree to which the lack of parasite conservation represents genuine opposition based on divergent conservation values, or to what extent it is the result of simply failing to appreciate the need to include parasites in conservation evaluations whether at the institutional or individual level.

The lack of parasite conservation actions could also be attributable to limited resources. More specifically, the literature on parasite conservation identifies several economic and technical challenges that act as barriers to more effective conservation practice. Conservationists have limited financial resources at their disposal with which to protect an increasing proportion of biodiversity (McCarthy et al., 2012). Parasite conservation will incur costs, such as the extra hosts required to support parasite populations or additional parasite extinction risk assessments (Dougherty et al., 2016; Moir et al., 2011), which may be unrealistic given available resources. Conversely, it has also been argued that relatively low‐cost actions to promote parasite conservation could significantly improve returns on investments in host conservation in terms of numbers of species protected (Moir et al., 2012). Another potential economic challenge for parasite conservation is that given parasites are normally viewed with aversion, fear, and apathy by the general public, the public is unlikely to support major expenditures or provide sizeable donations for parasite conservation (Kellert, 1993; Nichols & Gómez, 2011). Likewise, organizational funders are less likely to provide financial support for parasite conservation.

Current levels of parasite conservation are also determined by the poor availability of technical knowledge, experience, and resources. There is a general lack of practical guidelines and formal training for parasite conservation practice (Carlson et al., 2020). For instance, the current best practice guidelines on ex situ host conservation do not give sufficient guidance on how best to include parasite conservation (Lymbery & Smit, 2023). As such, conservationists lack the knowledge needed to turn general concerns about parasite biodiversity into actionable conservation targets (Gómez & Nichols, 2013). The importance of different social factors will vary for different parasite species, host species, and people. For example, people may have strong aversions to particular groups of parasites, and parasites with complex life cycles may be more difficult and expensive to conserve, both of which will render these groups of parasites less amenable to conservation. More research is needed to understand how sociocultural factors vary according to host and parasite species characteristics such that the feasibility of incorporating different parasite species into mainstream conservation practice can be assessed.

Conservation social science can be used to explore the social dimension of conservation in which human perceptions, attitudes, and values determine conservation outcomes for different species and ecosystems (Bennett et al., 2017). As such, parasite conservation will benefit from greater use of conservation social science to address the knowledge gap regarding the role of sociocultural factors in determining parasite conservation outcomes. We propose 3 main ways in which conservation social science can support parasite conservation efforts: to provide assessments of levels of awareness and support for parasite conservation, to improve understanding of different perspectives on parasite conservation, and to identify and mitigate sociocultural barriers to parasite conservation.

First, given the lack of study of human attitudes toward parasite conservation, there is a limited understanding of whether a lack of parasite conservation is the result of general ignorance toward parasite conservation issues or whether it may represent more principled objection to the concept. Surveys are a useful tool for establishing the basic views of a target population on a given topic (Sandbrook et al., 2019) and could be used to assess levels of awareness and objection to parasite conservation among practitioners or the public. Results from these studies will be useful in understanding the extent of social opposition to parasite conservation and in directing the focus of future outreach efforts.

Second, there is a paucity of understanding of the importance of the different sociocultural factors discussed above in determining parasite conservation outcomes. Qualitative and semiqualitative methods, including interviews and Q methodology, can produce in‐depth understandings of human perspectives, the knowledge of which will be useful in identifying particular barriers to parasite conservation and informing the design of more socially acceptable parasite conservation interventions (Drury et al., 2011; Zabala et al., 2018). This more detailed analysis can feed into collaborative methodologies from conservation social science, such as barrier workshops or fuzzy cognitive mapping, that allow researchers to work with conservation experts and other stakeholders to identify suitable conservation interventions that are supported by the involved groups (Rooney et al., 2023; Svolkinas et al., 2023).

Both these approaches emphasize identifying potential barriers to the implementation of conservation plans and how these might be navigated, which can support the development of tangibly applicable conservation actions. Taken together, this program of conservation social science will have an essential role in informing future conservation education practice, which has been identified as having a key role in future parasite conservation efforts (Carlson et al., 2020). The knowledge produced by social science research will be useful in understanding the scale of education and outreach efforts required to change attitudes toward parasite conservation and the specific content of these efforts, from general awareness raising of the importance of parasites to more targeted interventions, such as technical training for conservation professionals.

## REVISITING THE PHILOSOPHY OF PARASITE CONSERVATION

The philosophical basis for parasite conservation reflects the philosophy used to justify the conservation of free‐living biodiversity, drawing on both intrinsic and instrumental values to demonstrate why parasites are worth conserving. Since Windsor's (1990) initial provocation, these arguments have been partially successful—parasite conservation has entered into academic and public discourse (Nuwer, 2022), a small number of parasites have conservation plans (e.g. Kwak et al., 2019), and the first parasite has been featured on the IUCN Red List (Gerlach, 2014). Perhaps more significantly, there has yet to be any formal refutation of parasite conservation in the published literature; however, this does not mean that there is widespread support. In addition to the sociocultural factors and the inertia that often accompanies the uptake of new ideas, we propose that these objections could also result from weaknesses and contradictions in the philosophy currently underpinning parasite conservation. Philosophy, and particularly environmental ethics, provides useful analytical tools for evaluating different arguments and values around environmental issues such as conservation. Greater engagement with philosophical literature and methods will allow parasite conservationists to reevaluate their arguments, including their implicit assumptions and biases, to produce a more comprehensive parasite conservation philosophy. Such a philosophy will be invaluable on 2 counts: first, for motivating effective conservation practice inclusive of parasites and, second, for supporting conservation decision‐making processes in which multiple, potentially conflicting values must be evaluated against each other. We identified 2 problems with the current framing of parasite conservation philosophy that will need to be addressed if conservationists want to generate wider support for parasite conservation, and we suggest a third philosophical enquiry that will be useful to parasite conservation.

The first problem is that parasite conservationists have offered contradictory accounts of which parasite species are deserving of conservation. In their global parasite conservation plan, Carlson et al. (2020) explicitly exclude from conservation “parasites that are a known or suspected risk to human health, wellness, or livelihoods” and “parasites that threaten their host's conservation.” However, as argued by Lymbery and Smit (2023), these conditions unnecessarily exclude a sizeable proportion of parasite diversity from conservation. This approach is also overcautious relative to mainstream conservation practice, given the numerous examples of vertebrate species that pose a threat to human health and livelihoods and are considered conservation flagship species, including many wild carnivore species (Douglas & Veríssimo, 2013). Provided that risk is managed appropriately, with due consideration to who or what is at risk and the importance of all human life, it may be possible to conserve parasites that pose risks to people and their livelihoods. Similarly, given that parasites harm their hosts by definition, excluding parasites that threaten host conservation could be taken to mean all parasites of highly endangered hosts, which would include some of the most at‐risk parasite species. Rather than viewing parasites together as a common threat, more should be done to recognize that the risks associated with different parasites exist across a broad spectrum and that these risks are highly context specific, meaning that other actions can be done to mitigate risk rather than allowing species to become extinct.

A more fundamental contradiction with this approach is highlighted by examining the exclusions in relation to the justifications offered for parasite conservation. Carlson et al. (2020) argue that parasites should be conserved for their roles in ecosystem functioning and host evolution. Given that parasite‐induced sublethal impacts and host mortality are often an important part of these roles (Coltman et al., 1999; Hatcher et al., 2012), excluding parasites that threaten host conservation will mean excluding parasites that are important from an ecological and evolutionary standpoint. This will be the case whether the ecological importance of parasites is evaluated instrumentally (e.g., ecosystem services) or intrinsically (e.g., parasites derive value from being a component of intrinsically valuable ecosystems). Likewise, if the argument is made that species themselves are intrinsically valuable, there is little justification for excluding pathogenic parasites as these possess the same intrinsic value as their hosts and less harmful parasites (Lymbery & Smit, 2023). Instead of rejecting a number of species outright, a more nuanced approach to parasite conservation philosophy is needed, which accounts for the needs of human and animal hosts and acknowledges that harmful parasite species can also be ecologically important or possess intrinsic value. Such an approach will benefit from the integration of biological estimates of parasite health risks with conservation values that are clearly and consistently applied. Environmental ethics provides a framework for evaluating different factors, such as health risk and ecological importance, that can be used on a case‐by‐case basis to improve judgments of whether parasites should be conserved and how they can be best managed. Of particular importance is the need to balance short‐term interests around host welfare with the long‐term objective of preserving ecoevolutionary processes as part of holistic biodiversity conservation.

Another weakness in the current framing of parasite conservation is a reliance on conventional and value‐laden accounts of key conservation concepts, such as biodiversity, without making it sufficiently clear how or whether the values implicit in such concepts are embodied by parasite biodiversity. Proponents of parasite conservation have repeatedly argued that if the preservation of biodiversity is a central tenet of conservation, this should extend to parasite biodiversity, not just free‐living biodiversity (Pizzi, 2009; Windsor, 1990). At face value, this is a useful observation that highlights a common contradiction in conservation biology practice, whereby the stated goal of biodiversity protection is not realized due to the exclusion of certain elements of biodiversity including parasites. However, due to the fact that the term *biodiversity* is often used without precision or without appeal to a specific set of values (Lean & Maclaurin, 2016), the simple argument that biodiversity conservation should include parasites may not be sufficient to lead to a change in practice. Parasites may fall outside what people have in mind when using the term *biodiversity*; thus, the normative claim that biodiversity is good will not necessarily be automatically extended to parasites and be taken to mean that parasite biodiversity is good. More work is needed that explicitly focuses on demonstrating how the positive values conservationists associate with biodiversity are equally applicable to parasite diversity.

A more rigorous approach requires unpacking the values embedded in normative conservation claims—such as why biodiversity is good or why species are valuable—before evaluating each of these values against parasite diversity to present a more thoroughly evidence‐based argument for including parasites in conservation. One potential result of this approach is the identification of parasite species that have little conservation value and, similarly, specific values that will support the conservation prioritization of parasite biodiversity. Parasitologists and ecologists have already put this approach into practice by providing evidence of the different ways in which parasite biodiversity contributes to ecosystem function and structure, which has been used to support claims that parasites should be included in efforts to conserve biodiversity for the sake of ecosystem health (Wood & Johnson, 2015). Further work is needed to address the diverse array of values underpinning biodiversity conservation, including nonecological values, such as subjective intrinsic value or existence value, and how these different values might apply to parasites. Moreover, work is needed to link the values underpinning biodiversity conservation to individual parasite species to provide more tangible examples of the value of parasite conservation for practitioners and the public.

Another way in which philosophy can assist parasite conservation efforts is to re‐evaluate the framing of parasite conservation as being removed from and antagonistic to host conservation. This theoretical separation of host and parasite conservation has frequently led to negative outcomes for parasite biodiversity (Rózsa & Vas, 2015). Parasites are viewed as too costly, too harmful, or too difficult to conserve alongside their more charismatic host organisms (Gompper & Williams, 1998) or are simply not considered a relevant factor during host conservation (Stork & Lyal, 1993). Against this, parasitologists have pointed to the long‐term role of parasites in host evolution and the short‐term importance of parasites for host immune system development as evidence that parasite conservation can also have beneficial outcomes for host species and may even be considered an essential part of host conservation (Spencer & Zuk, 2016). Concurrently, approaches have been developed for minimizing the economic and health costs incurred by incorporating parasites into host conservation (Dougherty et al., 2016; Stringer & Linklater, 2014). We argue that the philosophy of biology can go further yet to overcome the false antagonism between parasite and host conservation, potentially opening up space for a new paradigm of conservation practice. Within the philosophy of biology, the concept of unitization deals with how biological entities are divided into distinct units, such as species and individuals.

A key concept in this field is that of the holobiont, which is used to describe a host and its associated microbiome as a singular evolutionary unit (Zilber‐Rosenberg & Rosenberg, 2008). This is supported by observations that symbiotic microbes play fundamental roles in host biology and ecology, including in host development, function, and evolution (Gilbert et al., 2012). As such, the term *holobiont conservation* is used to describe the conservation of a host and its associated microbiome, leading to improved outcomes for both parties (Carthey et al., 2020). Although the term *holobiont* as generally used excludes parasites, research into the holobiont has generated theory and concepts that are highly relevant to parasite conservation. Applying these concepts to host–parasite relationships raises the question to what extent can hosts be meaningfully conserved without maintaining their parasites? Arguably only a limited extent, given the significant coevolutionary relationships between hosts and parasites and the influence of parasites on host biology and ecology. As ecosystem restoration moves toward increasingly dynamic and process‐oriented approaches (Perino et al., 2019), we argue that single‐species conservation should move toward holobiont conservation in recognizing the importance of the full range of symbiotic relationships and the ecological and evolutionary processes they support.

**FIGURE 3:**
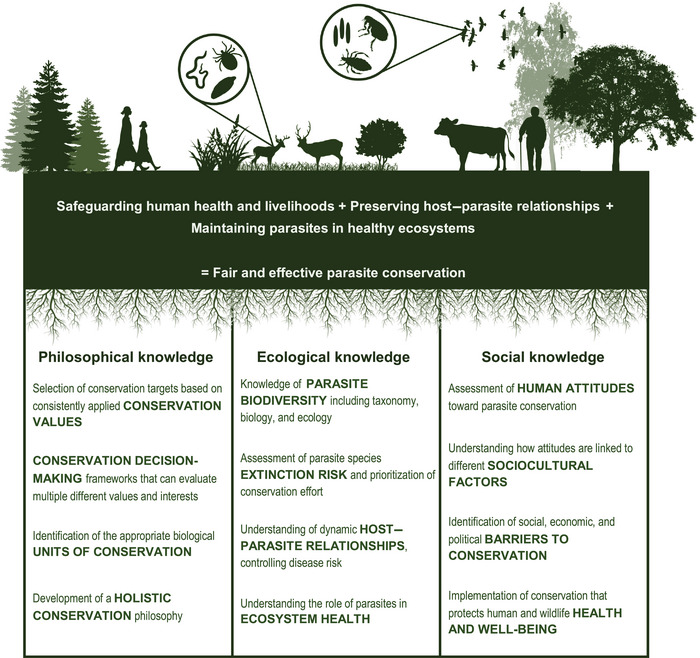
How the dimensions of philosophical, ecological, and social knowledge support effective and fair parasite conservation that balances the needs of humans, animal hosts, and parasite biodiversity.

More work is needed to explore how problems with current unitization concepts relate to host–parasite relationships and particularly the question of whether hosts can truly be considered as independent outside of the ecoevolutionary context of parasitic relationships. This work will create opportunities for new approaches to conservation, such as Kwak's (2018) concept of holistic conservation, which advocates for acknowledging the importance of symbiotic relationships for symbiotic partner species and ecosystems and designing conservation interventions that respect and preserve these important dynamics (Figure 3). More holistic approaches to species conservation could also close the gap between species‐level conservation and ecosystem‐level conservation, ensuring that species‐level interventions are attentive to the broader ecological context of a species’ existence.

## THE NEED FOR AN INTERDISCIPLINARY APPROACH FOR SUCCESSFUL PARASITE CONSERVATION

Parasite conservation lies at an exciting juncture. It is a growing field of conservation biology with the potential to enact protection for a large proportion of Earth's most at‐risk biodiversity. The recent creation of the IUCN Species Survival Commission Parasite Specialist Group (https://www.iucnparasites.com/) demonstrates the increasing credibility of the concept in academic and professional conservation circles. The main goals of this specialist group—to increase the number of IUCN assessments and conservation action plans for parasite species—are promising signs that progress will be made toward bridging taxonomic knowledge gaps and establishing the necessary technical frameworks for parasite conservation (Hopkins & Kwak, 2023). Although the concept of parasite conservation has a strong foothold within the disciplines of conservation biology and ecology, it lacks the broad professional or public awareness and support that are critical for more widespread conservation practice.

We have argued that 2 of the main barriers to more widespread parasite conservation are poor understanding of human perspectives on the issue and the need to refine current arguments for parasite conservation. Social factors are hugely important in determining conservation outcomes, and yet there has been no empirical study of human attitudes toward parasite conservation or how these are shaped by different values, perceptions, or knowledge of parasite biodiversity. Alongside conservation social science, we propose revisiting conservation philosophy to produce a more comprehensive and cohesive argument for parasite conservation. A sound philosophical foundation for parasite conservation is vital for demonstrating the value of parasite biodiversity and supporting conservation decision‐making processes. We have argued for a more nuanced philosophical approach to parasite conservation in which clearly laid out values are consistently applied and evaluated on a case‐by‐case basis.

In essence, effective parasite conservation requires an interdisciplinary approach. Only by bringing together knowledge, approaches, and tools from a range of disciplines (including ecology, conservation biology, parasitology, environmental ethics, philosophy of biology, and conservation social science) can conservationists develop a thorough understanding of the complex social–ecological systems of which parasites are part. This understanding will be necessary for designing and implementing parasite conservation interventions that balance the need for parasite and host conservation with the imperatives for protecting human health, well‐being, and livelihoods. Put simply, to prevent the loss of a significant proportion of the species that constitute the most common life history on Earth, parasite conservation has to make the jump from conservation research to conservation practice. The best chance of this happening will be when parasite conservation is supported by a broad disciplinary evidence base, bringing together a strong conservation philosophy with detailed knowledge of the ecological need and biological practicalities of parasite conservation, and a better understanding of the sociocultural barriers and requirements that will need to be managed and respected.

## AUTHOR CONTRIBUTIONS

*Conceptualization*: Timothy M. Brown, Alison M. Dunn, Ellen Clarke, Andrew A. Cunningham, Rupert J. Quinnell, and Simon J. Goodman. *Supervision*: Alison M. Dunn, Ellen Clarke, Andrew A. Cunningham, Rupert J. Quinnell, and Simon J. Goodman. *Writing—original draft*: Timothy M. Brown. *Writing—review and editing*: Timothy M. Brown, Alison M. Dunn, Ellen Clarke, Andrew A. Cunningham, Rupert J. Quinnell, and Simon J. Goodman.
